# A New Option for the Treatment of Intrahepatic Cholangiocarcinoma: Percutaneous Hepatic Perfusion with CHEMOSAT Delivery System

**DOI:** 10.3390/cells10010070

**Published:** 2021-01-05

**Authors:** Pier Francesco Ferrucci, Emilia Cocorocchio, Guido Bonomo, Gianluca Maria Varano, Paolo Della Vigna, Franco Orsi

**Affiliations:** 1 Tumor Biotherapy Unit, Department of Experimental Oncology, European Institute of Oncology, IRCCS, 20141 Milan, Italy; 2 Hematoncology Division, European Institute of Oncology, IRCCS, 20141 Milan, Italy; emilia.cocorocchio@ieo.it; 3 Interventional Radiology Division, European Institute of Oncology, IRCCS, 20141 Milan, Italy; guido.bonomo@ieo.it (G.B.); gianluca.varano@ieo.it (G.M.V.); paolo.dellavigna@ieo.it (P.D.V.); franco.orsi@ieo.it (F.O.)

**Keywords:** intrahepatic cholangiocarcinoma, liver metastasis, regional therapy, percutaneous hepatic perfusion

## Abstract

Liver metastases are a major management problem; since they occur in tumors of different origin, they are often multiple, difficult to visualize and can lie dormant for many years. Patients with liver metastases usually die of their disease, mostly due to liver failure, since systemic treatments are unable to eradicate micro-metastasis, and interventional loco-regional procedures cannot treat all existing ones. Cholangiocarcinoma (CCA) is the second most common primary liver tumor, showing a poor overall prognosis. When resection is not possible, treatment options include tumor-focused or local ablative therapy, organ-focused or regional therapy and systemic therapy. We reviewed available loco-regional therapeutic options, with particular focus on the CHEMOSAT^®^ Melphalan/Hepatic Delivery System (CS-HDS), which is uniquely positioned to perform a percutaneous hepatic perfusion (PHP), in order to treat the entire liver as a standalone or as complementary therapy. This system isolates the liver circulation, delivers a high concentration of chemotherapy (melphalan), filters most chemotherapy out of the blood and is a repeatable procedure. Most CS-HDS benefits are demonstrated in liver-predominant diseases, like liver metastasis from uveal melanoma (UM), hepatocarcinoma (HCC) and CCA. More than 650 procedures have been performed in Europe to date, mostly to treat liver metastases from UM. In CCA, experience is still limited, but retrospective analyses have been reported, while phase II and III studies are closed, waiting for results or ongoing.

## 1. Background

Choosing the therapeutic option for treating liver primaries or metastases may depend on factors such as histology, general patient conditions, characteristics of the disease (number, position and size of metastases), vascular anatomy and liver function, as well as timing in contemporary (synchronous) or later (metachrone) appearance, with respect to the primitive tumor.

For both primary and secondary hepatic tumors, surgical resection is considered the only curative therapeutic option [1]. This approach could imply the removal of even a large part of the liver, since, when healthy, it could regenerate. However, few patients are eligible to receive this procedure, since liver metastases often have a microscopic widespread dissemination and radicality is difficult to reach [2,3]. Moreover, disease may remain clinically silent until metastases are detected, because of multifocal spread and growth, causing progressive and rapid declining hepatic function.

Curative resection at this stage is no longer possible, and systemic therapies are considered the best option, allowing a usually time-limited control of the disease. In fact, to date, no systemic therapy has been shown to be decisive in the treatment of liver metastases, so that National Comprehensive Cancer Network (NCCN) guidelines, whenever possible, encourage the inclusion of patients in clinical trials [4,5,6]. On the other hand, the disease history could be different in those patients with liver predominant or liver only diseases such as hepatocarcinoma (HCC), uveal melanoma (UM) and cholangiocarcinoma (CCA). In particular, local therapies have been shown to be safe and effective in small studies of patients with unresectable CCA, which is the object of this review. Apart from surgery, such options include thermoablation, cryoablation, transarterial chemo-embolization (TACE) and transarterial radioembolization with yttrium-90 microspheres (TARE). These procedures emerged based on previous success in treating HCC and colorectal liver metastases [7]. However, treatment of unresectable CCA remains an unmet need, and there are no established first-line loco-regional options available [4,5].

Whole-organ-focused strategies had been proposed and performed, while more are under evaluation, in order to extend benefits addressing those complaints [8,9,10,11,12,13,14,15,16,17,18,19,20,21,22,23,24,25]. In particular, the CHEMOSAT^®^ Melphalan/Hepatic Delivery System (CS-HDS) product is designed to perfuse the entire liver with a chemotherapeutic agent (melphalan hydrochloride), with simultaneous extra-corporeal filtration of the hepatic venous blood, in order to remove the drug before it is returned to the systemic circulation [26].

**Figure 1:**
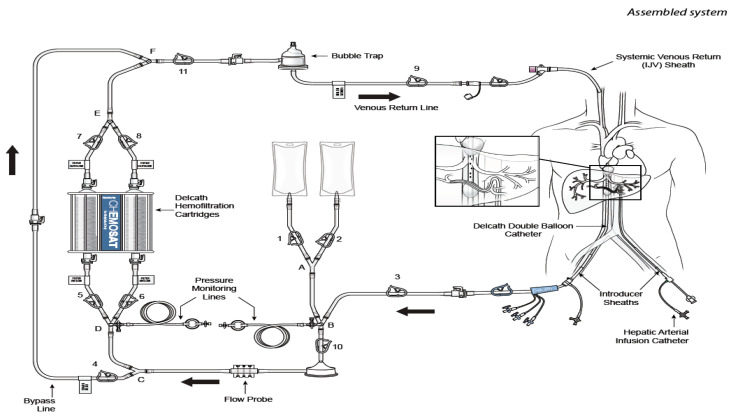
Cartoon reproducing the CHEMOSAT^®^ percutaneous hepatic perfusion (CS-PHP) Hemofiltration Circuit with extracorporeal circulation (Delcath Systems permission). Effective treatment of non-resectable primary and secondary liver tumors remains a major challenge in interdisciplinary oncology, with the general objective of expanding clinician roles towards loco-regional treatments and, in particular, to earn patients some time in terms of longer disease control and good quality of life.

Unlike ablation or embolization therapies, which can treat a limited number of visible tumors, CS-HDS with percutaneous hepatic perfusion (PHP), permits the treatment of patients with diffuse dominant liver disease, i.e., tumors >5 cm in diameter, and numbering more than three. More importantly, the procedure does not result in interruption of blood supply to the healthy parts of the liver, thus bypassing the effects of non-target embolization seen in other focal therapies [26]. The direct injection of chemotherapeutics into the hepatic artery combined with selective capture and channeling of the venous hepatic flow into a hemofilter prior to its return to the patient, allows for the use of high local doses of melphalan while greatly reducing systemic exposure and toxicity (Figure 1). The relatively non-invasive nature of CS-HDS on hepatocytes also makes it amenable to be repeated on a regular basis, thus allowing multiple treatments [26].

## 2. CCA Biology, Tumoral Heterogeneity and Molecular Characterization

CCA is a heterogeneous group of rare, aggressive malignancies, but growing in incidence and mortality rates [1,2,3,4,5,6,27,28,29]. Although surgical resection improves survival, CCA is asymptomatic in early stages and is most often diagnosed in advanced stages when unresectable [1,4,27,30,31,32]. In this case, prognosis is very poor, with the vast majority of patients dying between 6 and 12 months after diagnosis [33]. In particular, five-year survival rate following intrahepatic CCA (iCCA) resection remain between 22% and 44%, being lymph node metastasis, ≥5 cm tumor size and lymphovascular/perineural invasion, independent predictors of survival [4,5,6]. Similarly, the overall survival (OS) for advanced biliary tract cancer patients receiving chemotherapy (2197 patients from 82 trials) has been reported to be 8.2 months, representing an unmet need to deal with [27].

Depending on the anatomical location of the primary tumor, CCA is classified into perihilar CCA (pCCA), distal CCA (dCCA) and intrahepatic CCA (iCCA). Among these, pCCA accounts for 50% to 60% of all cases of CCA, while dCCA accounts for 20% to 30% and iCCA for around 20%, being primary sclerosing cholangitis the most common risk factor at least for iCCA [1,27,32,33].

Efforts should be done in studying the tumor cell biology and the tumor microenvironment, in order to better understand their functional interplay, to identify specific signaling pathways crosstalk and, finally, unveil how they all significantly influence the evolution of the disease and its response/resistance to conventional and tailored therapies.

Recent studies on the characterization of the different CCA subtypes highlighted their extended heterogeneity from a morphological, histological, molecular and biological point of view. Heterogeneity is firstly related to tumoral cells ability to emerge at different sites of the biliary tree, showing diverse macroscopic or morphological cellular features [34]. In particular, cancer stem cells (SCs) appear to contribute significantly to sustain this scenario, allowing the proposal and development of new classification based on the cell of origin as the first cell to acquire a pathogenic mutation [35]. Two SCs niches have been described within the liver: the Canals of Hering containing human Hepatic SCs and the Intra-Hepatic Peribiliary Glands composed of Biliary Tree SCs. These pluripotent cells can differentiate into hepatocytes and cholangiocytes, though possibly originating tumors with a whole range of phenotypes, varying from hepatocellular to biliary differentiation patterns. Moreover, stem cell self-renewal regulation, involve multiple signaling pathways associated with oncogenesis, including the Notch, Sonic hedgehog and Wnt signaling and their impairment has been shown to impact on the poorer prognosis and higher recurrence rate after CCA surgical resection and treatment [34,35].

At a genomic level, primary liver cancer heterogeneity is linked to a complex mutational landscape with molecular and biological variations that also contribute to disease development, drug resistance and tumor relapse following therapy, thus influencing significantly patient’s outcomes [36].

A recent review by Liu et al., identify two situations with different but integrated impact on disease pathogenesis. Getting into details, altered genotype and phenotype induced by diverse etiological and environmental factors influence intertumor heterogeneity, while genomic and biological variations gained by a single tumor cell due to evolution under multiple microenvironments’ pressures are included in the so-called intratumor heterogeneity [37].

In iCCA, most of the current understanding is limited to intertumor heterogeneity, allowing for molecular subclassification of patients based on their specific gene profiling in order to facilitate targeted therapy choices. A previous, more functional evaluation had revealed stable intratumor molecular subtypes of iCCA, allowing for categorization into two major subclasses linked to patients’ outcomes: the proliferation subtype and the inflammation subtype [37,38,39].

On the other hand, single cell transcriptomic datasets are a valuable resource to dissect cellular diversity and intercellular crosstalk, showing chemokines modulated interactions between cancer cells, T-cells and cancer-associated fibroblasts [38]. Different non-genomic events, including histone modifications, DNA hypo- or hyper- methylation, non-coding RNAs, and transcriptional regulators, by disrupting the epigenome, are able to contribute to intratumor heterogeneity, through their impact on regulating the spatial chromatin organization and altering the transcriptome [40,41].

Using different approaches, like in situ imaging, single cell and bulk tumor sequencing, is becoming possible to catch the compositional cell’s subclones within each tumor. Intratumor heterogeneity can be quantified by Shannon diversity index and compositional subclones can be categorized by using phylogenetic relationship. In vivo PDTX, in vitro PDTC and spheroid formation are the preclinically relevant best-fit models, which mostly recapture and preserve the compositional heterogeneity within a tumor and can be used for drug screenings [42].

However, all those analyses may not capture the whole tumor spectrum, while only the full understanding of the link between intertumor and intratumor heterogeneity will help improving subclassification and treatment stratification of patients [36]. For example, when interpreting the importance of intratumor genomic heterogeneity, a step forward is the development of a genome-axis evolution model, which sustain that multiple gene modifications could increase the adaptive function of a cell and influence its survival [43].

Recent efforts in molecular profiling have being able to identify actionable targets, leading to the emergence of promising novel therapies for treating CCA as reported in Section 3.2 [44]. However, finding specific CCA treatments is challenging, again, because of the marked heterogeneity of this disease, being only small percentages of patients responsive to inhibitors targeting genes mutations or aberrations.

Finally, microenvironment heterogeneity, considered as a novel hallmark of cancer, in general have a deep influence on tumor development and therapeutic efficacy, through the regulation of the immune editing balance. The direct interaction between tumor cells and heterogeneous stromal cells induces immune-regulating cytokine secretion and promotes intratumor heterogeneity, thus favoring immune-suppression [44,45]. CCA microenvironment show different gene expression profiles for immune checkpoint pathways, and though, effects of immunotherapy may be limited to small numbers of patients. On the other hand, there is great interest in combination therapies, where immune checkpoint blockade is coupled with existing or experimental drugs or even with loco-regional treatments [46,47].

**Figure 2:**
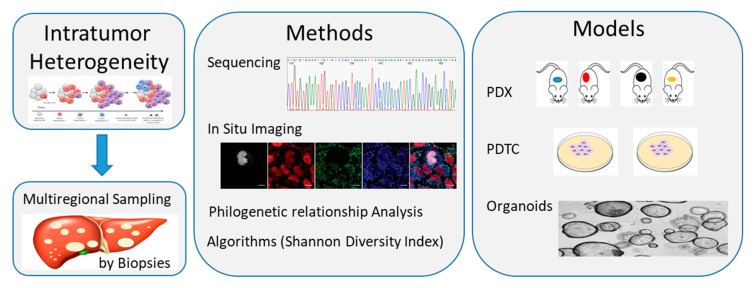
Schematic diagram showing methods and models available to study intratumor heterogeneity of cholangiocarcinoma (CCA). Multiregional sampling of the tumor through multiple biopsies, followed by single cell and bulk tumor sequencing or in situ imaging, can allow to catch the compositional subclones within each tumor. Data can be quantified by diverse algorithms, like the Shannon Diversity Index, and categorized by using Phylogenetic relationship analysis. Preclinical studies on in vivo tumor xenograft and in vitro cell cultures (cell layers or spheroids/organoids) models could allow us to recapture and maintain the compositional heterogeneity within a tumor and use it for drug screening. PDTC, patient-derived tumor cell; PDX, patient-derived tumor xenograft.

All these studies are only at the beginning in CCA and the heterogeneity of this cancer further hampers advancement in order to develop personalized treatments for our patients (Figure 2).

## 3. Current Treatment Options for Locally Advanced or Metastatic iCCA

### 3.1. Standard of Treatment

Although the only curative treatment for CCA presently is surgical resection, difficulties in diagnosing the disease in early stages, ultimately results in only 10% to 35% of patients with CCA eligible for resection [4,5,6,48,49]. Further, recurrence is common and occur in approximately 61% of patients at a median follow-up of 12.4 months [50].

The role of adjuvant chemotherapy is meaningful and was investigated in the phase III BILCAP trial, where 447 patients with resected CCA were randomized to receive adjuvant capecitabine or observation [51]. Results showed a significant advantage in median overall survival (OS) for patients receiving adjuvant capecitabine in respect to those undergoing observation (51.1 versus 36.4 months), [51]. Moreover, although gemcitabine combined with platinum compounds (especially cisplatin) is the current standard therapy in the metastatic setting, when adjuvant, its effect on OS seems comparable to other regimens inducing lower toxicity [5,6,52]. For these reasons, current National Comprehensive Cancer Network (NCCN) clinical practices recommend adjuvant chemotherapy with capecitabine for patients with resected biliary tract cancers [4].

Although liver transplantation is considered a feasible option for iCCA, the reported five-year survival varies greatly, depending on the study, from 10% to 70%, being affected by many biases [6]. For this reason, it is not included in the NCCN guidelines, nor in those published by the International Liver Cancer Association (ILCA) [4,5].

Patients with unresectable iCCA or metastatic disease may receive systemic chemotherapy, regional treatments or best supportive care [53]. Anyway, the standard of care for first-line therapy has remained unchanged since 2010, following positive results from the phase III ABC-02 trial demonstrating survival benefit in patients receiving gemcitabine and cisplatin compared with gemcitabine alone [4,54]. This standard chemotherapy schedule with gemcitabine/platinum compounds was further confirmed in a pooled analysis of 104 chemotherapy trials with 2810 patients treated for advanced biliary tract carcinomas [52]. In particular, the combination showed 30% to 50% response rates compared to 20% to 40% with other agents. However, there was no significant impact on OS (median 15.2 and 13.9 months, respectively) or duration of response (median 8.1 and 6.6 months, respectively) [52,53,55].

More recently, the open-label single-arm phase II GAP trial investigated the addition of nab-paclitaxel to gemcitabine and cisplatin as first-line therapy for 60 patients with advanced biliary tract cancer. After a median follow-up of one year, median progression-free survival (PFS) was 11.8 months, and median OS was 19.2 months [56]. Results of the phase III trial are awaited [57].

The standard of care for second-line systemic therapy for patients with unresectable CCA who have progressed on first-line therapy was recently established by the phase III ABC-06 trial [58]. In that study, 162 patients progressing on first-line gemcitabine and cisplatin were randomized to receive either active symptom control (ASC) together with FOLFOX (n = 81) or ASC alone (n = 81). The median OS was significantly greater in patients receiving chemotherapy compared with those receiving ASC alone (6.2 versus 5.3 months).

Finally, inclusion in a clinical trial represent an interesting opportunity and must always be considered for patients with evolving disease after standard treatments.

Numerous potentially targetable genetic driver alterations, including high microsatellite instability (MSI-H), isocitrate dehydrogenase (IDH)-1 and -2 mutations, and fibroblast growth factor receptor (FGFR) alterations, have recently been discovered and resumed for iCCA in Table 1 with ongoing related clinical trials [1,44,59,60,61,62,63,64,65,66,67,68,69,70,71,72].

In particular, immunotherapy with antiPD1 monoclonal antibody Pembrolizumab has demonstrated efficacy in treating MSI-H solid tumors [59,60], and was recently investigated in the multicohort phase Ib KEYNOTE-028 basket study on a total of 23 patients with biliary tract cancer. The overall response rate (ORR) in these patients was 17%, while the median OS and PFS observed were 6.2 and 1.8 months, respectively [61].

### 3.2. Local and Regional Treatment Strategies

Local and/or regional therapies using tumor-focused or organ-focused techniques have been included in the standard armamentarium, since they could work synergistically with systemic treatments [6,74,75]. As previously mentioned, these include percutaneous tumor ablations and different types of transarterial instillation of chemo- or radiotherapy, like hepatic arterial infusion (HAI), conventional drug- or radio-eluting embolization, and regional perfusion with focus on isolated PHP through CS-HDS.

Percutaneous tumor ablation may be achieved by thermoablation through radiofrequency (RFA) or microwaves (MW), laser or cryotherapy, as well as by the injection of chemicals such as ethanol, acetic acid or boiling saline [7,76,77]. Since these options are considered focal treatments that are adequate to treat the visible lesions, they are generally available only to patients with a limited number of small unresectable tumors. The few studies using RFA have shown less optimal results in iCCA patients than those achieved in HCC, being also associated with higher rate of adverse reactions.

Regional Hepatic Arterial Infusion (HAI) of chemotherapy is able to deliver higher local drug concentration to unresectable liver tumors with fewer significant systemic side effects, due to the first-pass effects of cytotoxic agents [75,78,79,80,81,82]. HAI has been shown to produce better response rates than systemic chemotherapy despite little impact on survival, mainly due to the development of extra-hepatic metastases. Although HAI has been used to treat patients with advanced and unresectable iCCA, it has not yet been evaluated in prospective randomized clinical trials. In this line, selective Transarterial Embolization and Transcatheter Arterial Chemo-Embolization (TACE) represent other useful options, being able to deliver and concentrate chemotherapy in the metastatic tissue, while sparing most of the healthy liver and other tissues of the body. It has been proven to be the most effective treatment strategy in terms of regression/stabilization of liver metastases, and even in terms of increased survival. Possible induced toxicity depends on the amount of the drugs reaching the systemic circle, thus limiting the administrable doses.

Selective Internal Radiation Therapy, or Transarterial radioembolization (TARE), is another minimally invasive procedure consisting of an infusion of radioactive microspheres loaded with yttrium-90 or lipiodil Iodium-131 directly into the vessels afferent to the tumor [83,84]. Although the procedure limits the damage to the general liver tissue, collateral toxicity of the tumor surrounding healthy cells, due to regional blood supply cutoff, remains a concern.

Organ-focused treatments are regional delivery techniques developed to reach macro- and micro-metastatic disease, as they diffuse into the liver and control possible collateral effects [8,9,10,11,12,13,14,15,16,17,18,19,20,21,22,23,24,25,26]. Through the complete exclusion of the liver from systemic circulation and its integration into an independent extracorporeal circuit, the whole organ can be perfused with chemotherapy at very high doses, higher than those obtainable through systemic administration with negligible systemic toxicity. They include a surgical hepatic perfusion (SHP) and CHEMOSAT percutaneous liver perfusion (CS-PHP).

SHP is an open abdomen surgery in which the circulation of the liver is isolated by placing cannulas in the portal vein, hepatic artery and retrohepatic inferior vena cava, in order to route the blood from these vessels into an extra-corporeal circuit. The liver perfusate is used to deliver antineoplastic drugs at high dosages. Although only a few centers have reported substantial experience with these procedures, it appears to be effective even in advanced tumors or tumors refractory to other therapies.

SHP resembles for goals and results the second option, CS-PHP, but it is not repeatable and maintains all the risks related to long and demanding surgery. Instead, CS-PHP is a minimally invasive, repeatable regional therapy for non-resectable hepatic metastases. This system of catheters and filters isolates the hepatic venous blood from the systemic circulation, allowing for the delivery of high-dose melphalan hydrochloride (L-PAM) to the hepatic artery. Systemic exposure to the drug is reduced by filtering the effluent hepatic venous blood before it is returned to the circulation. L-PAM, a non-specific bifunctional alkylating agent, was selected as the active chemotherapeutic agent for the formal clinical trial program of CS-PHP based on several observations. Firstly, there is in vitro evidence to suggest that L-PAM is effective in killing HCC cell lines. Secondly, it does not cause significant liver toxicity even when given at doses used for myeloablation in the clinic. Thirdly, L-PAM delivered by operative isolated hepatic perfusion has previously shown efficacy in patients with hepatic metastases from a variety of cancers, including melanoma, colorectal cancer, hepatocellular carcinoma and neuroendocrine tumors. Lastly, L-PAM is widely available and relatively inexpensive, making it an accessible choice for clinics around the world. The feasibility of CS-PHP has been shown in several studies of patients with unresectable hepatic metastases or primary hepatic cancer.

## 4. CS-PHP Clinical Development

### 4.1. Overview of Previous Studies in Tumors with Liver Metastases

A formal clinical trial program for CS-HDS is ongoing, and phase I [14], phase II [85] and phase III studies [15,16] have been conducted on different diseases and are now completed. In particular, a total of 153 patients with unresectable liver tumors have received L-PAM with CS-HDS in those three clinical studies. Additionally, patients have been treated in Compassionate Use and in the Expanded Access Program in the US, or received the treatment outside a clinical trial in the European Union, where the PHP system has been on the market since February 2012.

Most of the clinical experience has been done in ocular melanoma, iCCA and HCC due to the specific characteristics of the evolution of these diseases in the metastatic phase (Table 2) [14,15,16,85,86,87,88,89,90,91].

### 4.2. Safety Experience with CS/HDS Treatment

Safety data are available for 153 patients included in the clinical studies previously described [14,15,16,85]. They all were started on a melphalan dose of 3.0 mg/kg. Treatments were administered every four to eight weeks, with patients receiving a median of three cycles of treatment in the phase III study and median of two cycles in both the phase I and the phase II studies.

Adverse events (AEs) have been categorized into two periods to distinguish between those observed in the peri-operative period and those occurred subsequently in the post-operative period. The peri-procedure period is defined as the time between the date of planned procedure plus three days. The post-procedure period is defined as the end of the peri-procedure period (three days) extending to the start of the next procedure or 30 days post-treatment—whichever is later.

**Figure 3:**
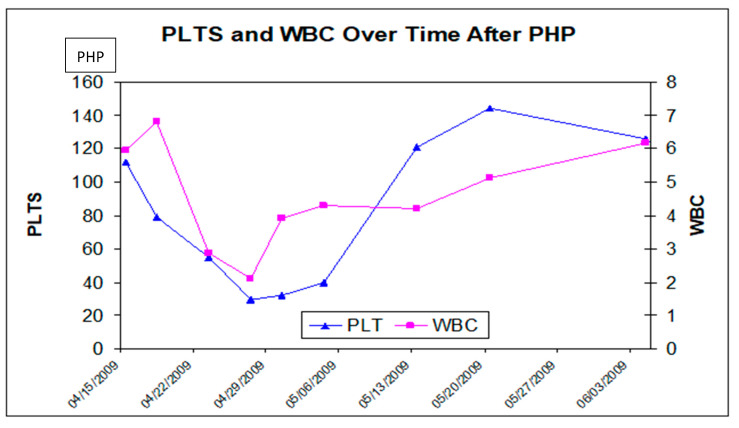
Peri- and post-operative hematological toxicity in an explanatory PHP case. The prolonged partial thromboplastin time (aPTT) was an intended outcome from heparin anticoagulation required to perform hemofiltration. Plasma proteins, such as albumin and certain coagulation factors, were also removed by the filters, but they were typically corrected by the administration of fresh frozen plasma and/or cryoprecipitate at the end of the procedure.

The most frequent AEs during the peri-procedure period were the direct consequences of the procedure, including the need for systemic anticoagulation, hemofiltration and embolization. Frequent peri-procedural AEs included a decrease in platelet count, hemoglobin levels, lymphocytes, blood albumin, blood calcium and blood potassium; prolongation of the activated partial thromboplastin time (aPTT); and increase of the international normalized ratio (INR), the aspartate aminotransferase (AST), the alanine aminotransferase (ALT) and the blood bilirubin. The decreased platelet counts and hemoglobin were a consequence of platelet and red-blood-cell sequestration by the filters and were managed with transfusions when clinically indicated (Figure 3).

The most common AEs reported in the post-procedure period were related to myelosuppression, including thrombocytopenia, neutropenia and anemia, which, in general, were also the most frequent serious adverse events (SAEs) (described as shown in Table 3).

During the history of the CS-PHP development, five treatment related deaths (due to hepatic failure, GI perforation, GI hemorrhage and sepsis,) were observed and reported. However, complications in the post-procedure period has since led to some modification of the patient’s screening procedure, the melphalan/HDS treatment protocol and the subsequent patient management. These changes included a series of measures, like the exclusion of patients with >50% tumor involvement who are obese or received prior Whipple’s procedure, the application of nitroglycerin to relieve hepatic arterial spasm during melphalan administration (if observed), the complete imaging to exclude hemorrhagic risks in patients with central nervous system lesions, and the prophylactic use of granulocyte growth factors after each procedure. Since these risk-mitigation guidelines were implemented, no recurrence of fatal complication has been observed [89,91].

### 4.3. CS-PHP Efficacy in Cholangiocarcinoma

Low numbers’ experience is available on CCA patients (mainly included in small phase I and II trials or in multi-center retrospective data collections), but the data showed interesting efficacy signals [14,85,86,87,88,89,90,91]. The first report to acknowledge is from a two centers analysis that included 14 consecutive patients with unresectable hepatic metastases from solid tumors [89]. In this sequence, one single patient with biliary tract adenocarcinoma was enrolled and experienced a complete response to treatment with melphalan/HDS according to response evaluation criteria in solid tumors (RECIST) criteria. This young patient (44 years old at diagnosis, on March 2011) had multiple liver metastases up to 71 mm in diameter and was heavily pretreated at time of PHP, having progressed to systemic oxaliplatin/gemcitabine and multiple HAI cycles. She received only one CS-HDS procedure in December 2012, experiencing a G4 hematological toxicity with sepsis, from which she recovered after two months. Complete response was achieved after three months from the procedure and is still lasting since the patient is alive after almost eight years from liver perfusion (almost 10 years from initial diagnosis of liver metastatic CCA), without needing any further therapy for her cancer. Unfortunately, she developed a sclerosing cholangitis and received a liver transplantation on November 2019, seven years after the CS-HDS procedure. Recently she had a graft versus host disease and a COVID-19 infection, from which she recovers, being now in good general condition with no evidence of active cancer or complications (our unpublished data).

Two patients were enrolled in the primary hepatic malignancy cohort of a Phase II study: Within this cohort, five had histologically confirmed HCC and two had iCCA (Clinical Study Report 04-C-0273) [85]. The primary efficacy endpoint of the study was hepatic objective response rate (hORR) and duration of hORR. The secondary efficacy endpoints were hepatic progression free survival (hPFS) and OS. The initial dose of L-PAM was 3 mg/kg ideal body weight (IBW). Of the two with iCCA, patient 001-001 was a 62-year-old white female who received four CS-HDS treatments, while patient 001-007 was a 48-year-old white male who received two CS-HDS treatments. Both of them were withdrew from treatment due to extra-hepatic disease progression and died 10 months and 8 months after having started CS-HDS treatment, respectively. The acceptable safety and tolerability of CS-HDS treatments was supported by the observation that multiple treatments were administered to the patients with no new safety signals emerging [85].

Another phase II trial was designed and partially performed to investigate the role of CS-HDS in HCC and iCCA patients; the results are still awaited. This was a two-arm, open label, multi-center phase II study to evaluate the efficacy and safety of CS-HDS in patients with unresectable HCC or iCCA confined to the liver (Clinical Study Protocol PHP-HCC-202). A total of 42 evaluable patients, 31 with HCC and 11 with CCA, were planned to be enrolled into this two-stage, two-cohort study. For the CCA cohort, if there were ≤3 responses in the 11 patients, therapy was not considered worthy of further investigation. The main objectives of the study were to estimate the ORR, the PFS and the safety of patients receiving the CS-HDS treatment, while exploratory analyses were focused on characterizing the systemic exposure of melphalan at selected study sites [91].

**Figure 4:**
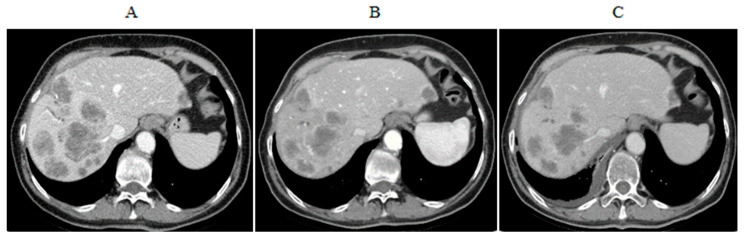
CT scans of the European Institute of Oncology (EIO) responding patient with liver metastasis from iCCA included in the phase II trial. She received two cycles of CS-PHP and achieved a partial response with reduction in the number and dimension of the lesions. Pictures show (**A**) pretreatment, (**B**) after first PHP and (**C**) after second PHP.

At the European Institute of Oncology (EIO), we enrolled four iCCA patients in this study (one screening failure), and three received two PHP procedures, each as planned, with good tolerability. Results showed one stabilization of disease (SD) lasting eight months from the first procedure, one PR lasting 16 months from the first procedure and one progressive disease (PD) just after the second PHP treatment (Figure 4 and EIO unpublished data).

Kirstein and colleagues reported a retrospective analysis on 29 patients with liver metastasis from different cancers, all treated with CS-HDS [88]. Among them, five patients had CCA and where heavily pretreated with previous systemic and loco-regional treatments. ORR was 19.2%, with SD after just one PHP treatment in up to 55.2% patients. Disease control rate was 74.4% with a median PFS of 117 days, median hPFS of 135 days and median OS of eight months from first PHP. Two patients had an ongoing SD for 372 and 454 days, respectively.

Recently another retrospective study reviewed data from 15 patients with unresectable iCCA treated with CS-HDS in nine different hospitals throughout Europe [90]. Overall response rates were assessed according to response evaluation criteria in solid tumors (RECIST 1.1), while OS, PFS and hPFS were analyzed using the Kaplan–Meier estimation. AEs and toxicity were also evaluated in order to deeply explore the feasibility of the procedure in this setting. A total of 26 CS-HDS procedures were performed on those 15 patients and results showed an ORR of 20%, while disease control was achieved in 53% of patients just after the first PHP. In particular, one patient (7%) experienced a CR, two patients (13%) had a PR, while eight patients (53%) had a stabilization of disease as a best response. Three patients (20%) developed a PD and one died before performing a follow-up imaging at 46 days after PHP treatment due to sepsis and liver failure. Five of the patients with SD received a second treatment resulting in one PR (20%), one PD (20%) and three SD (60%). Subsequently, three more procedures were performed in two of those patients who had a SD during long-term follow-up. In these cases, median time between first diagnosis and first PHP was 17.2 months (range 2.0–41.5 months); median time between first and second PHP was 3.2 months (range 2.1–4.2 months), median PFS and hPFS were 122 and 131 days, respectively. Median OS was 26.9 months from initial diagnosis and 7.6 months from first PHP. Interestingly, patients with liver-only disease showed a significantly longer median OS compared to patients with loco-regional lymph node metastases (12.9 vs. 4.8 months, respectively; *p* < 0.01). Hematological toxicity was common, mainly occurring in the peri-procedural period, but manageable as expected and no AEs of Grade 3 or 4 occurred post treatments [85,86,87,88,89,90,91].

Finally, a randomized, controlled, phase III study to compare the efficacy, safety and pharmacokinetics of melphalan/high doses (L-PAM/HDS) treatment given sequentially following cisplatin/gemcitabine versus cisplatin/gemcitabine, is planned, but currently in stand-by. Primary objective of this study is OS, secondary objectives are PFS and objective response rate (ORR). Patients are to be enrolled in a first phase (62 patients randomized to L-PAM/HDS and 62 patient remain on standard treatment). The second phase will conclude enrollment at individuated doses with an additional 146 patients to be enrolled (73 each arm) in approximately 40 sites in Europe and US.

## 5. Discussion

Although surgery is the primary standard treatment option for iCCA, resection is only possible in less than one-third of patients, at diagnosis, due to the presence of advanced disease. Liver metastases are then treated with systemic and regional treatments, trying to combine synergistic effects, but the results are often poor and liver failure is the limiting event in most of the patients.

Preventing or delaying liver failure due to progressive disease is an unmet need, and extending the window of opportunity, thus maintaining a good quality of life, is an objective to be focused on when approaching the treatment of this disease.

CS-HDS is a minimally invasive technique which delivers high doses of chemotherapy directly to tumors in the liver, while limiting systemic toxicity through hemofiltration of the hepatic venous blood. It is a palliative treatment, to be compared with other loco-regional procedures in terms of toxicity and efficacy, having the objective to reach a better and longer tumor control in patients with few options and liver evolving disease.

Standardization of the PHP technique allowed for its wider utilization, offering an additional strategy for the treatment of unresectable primary or secondary intrahepatic malignancies.

In this manuscript, we reviewed available data on the safety and efficacy of CS-HDS in the treatment of iCCA. Importantly, results are summarized mostly for patients being heavily pretreated, who received PHP as a last option in their therapeutic armamentarium.

Reported cases were treated in a wide period of observation, during which the procedure has been further developed, allowing for better selection of patients, identification of better supportive cares and, last but not least, to the use of new generation of filtration systems (GEN2 vs. GEN1). This evolution provided definitive evidence of the improved toxicity profile, while maintaining the same level of efficacy. In fact, side effects are now tolerable, manageable and comparable to other conventional systemic or localized strategies, especially when procedures are performed by skilled multidisciplinary teams.

Hematological toxicity requires a lower transfusional support in respect to the initial utilization of GEN1 filters and only few cases of leuco-neutropenia, since the addition of a short growth factors course after each procedure were described. Other kinds of systemic toxicity, such as fatigue or epigastric/abdominal pain, were rapidly recovered and did not influence the possibility to perform other standard treatments as for best clinician choices, in case of progressive disease.

Despite low numbers of CS-HDS-treated iCCA patients and the limited value of retrospective trials, significant results were achieved after patients’ considerable pretreatment with chemotherapy and other loco-regional approaches. Moreover, CS-HDS was confirmed to be a safe procedure that provides promising response rates and survival data in metastatic patients, especially in liver-localized disease.

Unlike other regional and local therapies, CS-HDS is able to treat the whole liver, including large (>5 cm) tumors, as well as disseminated metastases and co-existing occult or microscopic lesions. In the absence of valid alternatives this palliative option should be considered in the attempt to realize a longer and efficacious control of the disease, giving to our patients the opportunity to expand their life expectations maintaining a good quality of life.

Considering that the median OS in patients with liver metastases from CCA is generally very low, ranging from six to eight months irrespective of the treatment chosen, we conclude that the reviewed experience with PHP seems to allow for a longer control of the disease. In particular, CS-HDS could be used earlier in the treatment sequence, in order to gain time before switching to systemic treatment when disease evolves outside of the liver. Moreover, combination or sequence strategies with chemotherapy could be explored in order to obtain a better and longer control at a systemic level, maintaining a valuable quality of life.

In conclusion, when disease is limited to the liver, CS-HDS should be taken into consideration by an experienced multidisciplinary team, in order to add an effective weapon to our limited options’ armamentarium.

## Figures and Tables

**Table 1:** Genetic variants in CCA as possible effective therapeutic targets and ongoing clinical trials.

Target	Prevalence	Trial/Drug
TP53 mutation	27% iCCA; 40% pCCA/dCCA	
KRAS mutation	22% iCCA; 42% pCCA/dCCA	
ROS1 rearrangement	8% to 9%	
MSI-H	14% to 18% iCCA	KEYNOTE-028/Pembrolizumab [59,60,61]
TMB-H	6% to 12% iCCA	[73]
CDKN2A mutation	47% iCCA	
IDH1/IDH2 mutation	25% iCCA	ClarIDHy/Ivosidenib [63]
FGFR2	10% to 16% iCCA	FIGHT-202 and -203/Pemigatinib [64,65,66,67] Infigratinib [68] Debio1347, Derazantinib, Erdafitinib, Futibatinib [69,70,71,72]
EGFR overexpression	16% iCCA	
MET amplification	2% iCCA	

Notes: pCCA, perihilar CCA; dCCA, distal CCA; iCCA, intrahepatic CCA.

**Table 2:** EU experience with CS-PHP at December 2019. Total procedures performed in all cancers and in CCA are in bold. In particular, a total of 76 CS-PHP procedures were performed on 42 CCA patients mainly included in clinical trials or in standard indication.

Liver Tumor Type	No. of Patients	No. of Treatments
Ocular Melanoma	221	489
**Cholangiocarcinoma**	**42**	**76**
Colorectal	15	24
Hepatocellular carcinoma	13	20
Pancreatic	7	14
Neuroendocrine	7	12
Cutaneous Melanoma	6	9
Breast	5	11
Others	5	5
**TOTAL**	**321**	**660**

**Table 3:** Grade 3/4 and Grade 5 treatment related hematological and hepatic toxicity as experienced by a selection of 55 patients who performed 160 treatments within clinical trials or following standard indication.

Hematological Toxicity on 160 PHPs	Grade 3/4, n (%)	Grade 5, (%)
Neutropenia	102 (63.7)	
Thrombocytopenia	119 (74.4)	
Anemia	75 (46.9)	
Neutropenic Sepsis		2 (1.25)
**Hepatic Toxicity on 160 PHPs**		
Elevated AST	23 (14.4)	
Elevated ALT	11 (6.9)	
Elevated Bilirubinemia	16 (10)	
Elevated ALP	9 (5.6)	
Liver Failure		1 (0.62)

Notes: AST, aspartate aminotransferase; ALT, the alanine aminotransferase; ALP, Alkaline Phosphatase.

## Data Availability

Data are available in our EIO digital records for the single patient’s reported history. Other data have been reviewed from published articles referred to clinical studies.
